# The Characterization of Disease Severity Associated IgG Subclasses Response in COVID-19 Patients

**DOI:** 10.3389/fimmu.2021.632814

**Published:** 2021-03-04

**Authors:** Huanle Luo, Tingting Jia, Jiamin Chen, Shike Zeng, Zengzhao Qiu, Shu Wu, Xu Li, Yuxuan Lei, Xin Wang, Weihua Wu, Renli Zhang, Xuan Zou, Tiejian Feng, Ruxia Ding, Yue Zhang, Yao-Qing Chen, Caijun Sun, Tian Wang, Shisong Fang, Yuelong Shu

**Affiliations:** ^1^School of Public Health (Shenzhen), Sun Yat-sen University, Shenzhen, China; ^2^Key Laboratory of Tropical Disease Control, Ministry of Education, Sun Yat-sen University, Guangzhou, China; ^3^Shenzhen Center for Disease Control and Prevention, Shenzhen, China; ^4^Division of HIV/AIDS and Sex-Transmitted Virus Vaccines, Institute for Biological Product Control, National Institutes for Food and Drug Control (NIFDC), Beijing, China; ^5^Department of Microbiology and Immunology, University of Texas Medical Branch, Galveston, TX, United States; ^6^Department of Pathology, University of Texas Medical Branch, Galveston, TX, United States; ^7^Institute for Human Infections and Immunity, University of Texas Medical Branch, Galveston, TX, United States

**Keywords:** SARS-CoV-2, COVID-19, host immune response, antibody response, cytokine production, disease severity, IgG subclasses

## Abstract

Increasing evidence suggests that dysregulated immune responses are associated with the clinical outcome of coronavirus disease 2019 (COVID-19). Nucleocapsid protein (NP)-, spike (S)-, receptor binding domain (RBD)- specific immunoglobulin (Ig) isotypes, IgG subclasses and neutralizing antibody (NAb) were analyzed in 123 serum from 63 hospitalized patients with severe, moderate, mild or asymptomatic COVID-19. Mild to modest correlations were found between disease severity and antigen specific IgG subclasses in serum, of which IgG1 and IgG3 were negatively associated with viral load in nasopharyngeal swab. Multiple cytokines were significantly related with antigen-specific Ig isotypes and IgG subclasses, and IL-1β was positively correlated with most antibodies. Furthermore, the old patients (≥ 60 years old) had higher levels of chemokines, increased NAb activities and SARS-CoV-2 specific IgG1, and IgG3 responses and compromised T cell responses compared to the young patients (≤ 18 years old), which are related with more severe cases. Higher IgG1 and IgG3 were found in COVID-19 patients with comorbidities while biological sex had no effect on IgG subclasses. Overall, we have identified diseases severity was related to higher antibodies, of which IgG subclasses had weakly negative correlation with viral load, and cytokines were significantly associated with antibody response. Further, advancing age and comorbidities had obvious effect on IgG1 and IgG3.

## Introduction

Severe acute respiratory syndrome-coronavirus 2 (SARS-CoV-2), is a newly emerged coronavirus causing huge causality of human (1). As of 23 November 2020, SARS-CoV-2 has spread to 216 countries and regions, causing more than 58 million cases including 1,385,218 confirmed deaths (https://www.who.int/emergencies/diseases/novel-coronavirus-2019/situation-reports/). World Health Organization (WHO) officially designated the disease caused by SARS-CoV-2 as coronavirus disease 2019 (COVID-19), the clinical manifestations of COVID-19 include fever, dry cough, tiredness, dyspnea, myalgia, fatigue, and even severe respiratory illness with pneumonia as the most common complications. It is estimated that up to 20% COVID-19 cases developing to acute respiratory distress syndrome (ARDS) were associated with elevated levels of plasma cytokines (2–4). Hyperinflammation has been reported to be involved in coronavirus pathogenesis. For example, SARS-CoV and Middle East respiratory syndrome-coronavirus (MERS-CoV) both induce aberrant pro-inflammatory cytokine and chemokine response, resulting in acute lung injury (ALI), and ARDS (5, 6). During SARS-CoV-2 infection, elevated IL-6, TNF-α, IL-1β, and inflammatory chemokines including IL-8, IP-10 were correlated with the disease severity and the corresponding agonists were used as therapeutic options for COVID-19 (4, 7). Importantly, IL-6, TNF-α have been reported to predict the severity of COVID-19 patients (8). Besides, humoral response is considered being involved largely in host immune reaction during microbial infection. The multi-isotype antibodies in serum include IgA, IgD, IgG, IgM, and IgE, of which IgG is the most abundant, while IgD and IgE are extremely scarce (9). Thus, assessment of antibody responses has been focused on the titers of IgA, IgG, and IgM (10). Four IgG subclasses in human have been identified which have more than 90% similar sequence, but distinct functions. For example, IgG antibody response to bacterial infection is mostly restricted to IgG2; while viral infections generally induce the IgG1 and IgG3 (11). The coronavirus infection in human commonly triggers various antibody responses, among of which neutralizing antibody (NAb) was broadly elicited in SARS-CoV or MERS-CoV patients and had anti-viral activities (12–16). During SARS-CoV-2 infection, virus-specific IgG and IgM responses were induced within the first 3 weeks after disease onset and were higher in the severe group than non-severe group (17). NAb isolated from COVID-19 patients was shown to reduce viral titers in animal models (18, 19), indicating the important role of NAb during control of SARS-CoV-2 infection. Other studies have demonstrated antibody responses including NAb increased with disease severity and were higher in the old COVID-19 men with comorbidities (20–22), suggesting the complicated feature of antibodies which is largely unknown. Although recent studies have described the immune responses in COVID-19 patients with different clinical outcomes (23–25), the associations of antibody with viral load, and with cytokines in the COVID-19 patients remain to be elucidated.

In this study, we analyzed 123 blood specimens collected from 63 individuals ranging from asymptomatic to severe COVID-19 patients admitted to hospital to characterize the immune response profile. Higher NAb, Ig isotypes including IgA, IgM and IgG, IgG subclasses against nucleocapsid protein (NP)-, spike (S)-, receptor binding domain (RBD) were found in the severe group than other groups. Antigen-specific IgG1 and IgG3 in serum were associated with disease severity and were negatively correlated with viral load in nasopharyngeal swab. The advancing age and comorbidities exhibited more obvious effect on IgG subclasses than total IgG, while biological sex had no effect on the IgG subclasses. Finally, the interplay between antibody response and cytokines was analyzed in detail to provide the full understanding of host immune response against SARS-CoV-2 infection.

## Materials and Methods

### Study Population and Ethics Statement

A total of 123 serum samples collected from 63 COVID-19 patients were obtained from Shenzhen Center for Disease Control and Prevention and stored at −80°C. Clinical classification of the patients follows the COVID-19 Prevention and Control Plan (5th edition) by clinicians. The subjects with symptoms were hospitalized COVID-19 patients (*N* = 57), 6 patients without symptom but with nucleic acid test positive were quarantined in hotel and 11 healthy subjects as controls including 4 males and 7 females, with an average age of 28 (ranging from 25 years old to 30 years old), were collected at baseline in a vaccine study performed in 2018-2019. Serum samples were heat inactivated at 56°C for 30 min before use. Supplementary Table 1 shows the detail demographics and baseline characteristics of these patients.

All the experiments were performed in compliance with and under the approval of the biomedical research ethics committee, the public health school (Shenzhen) of Sun Yat-sen University.

### Cell Lines

Huh-7 cells obtained from National Institutes for Food and Drug Control were maintained at 37°C with 5% CO_2_ in DMEM supplemented with 10% FBS, 100U /ml Penicillin-Streptomycin and 25 mM HEPES.

### Virus

SARS-CoV-2 pseudovirus with luciferase reporter gene used for neutralization assay was a kind gift from National Institutes for Food and Drug Control and stored at −80°C until detection.

### Proteins

SARS-CoV-2 Spike Protein (S1+S2) (cat# 40589-VO8B1), SARS-CoV-2 Spike RBD Protein (cat# 40592-V08B), SARS-CoV-2 Nucleocapsid Protein (cat# 40588-V08B) were purchased from Sino Biological (China).

### Viral Load Calculation

Viral RNAs were extracted from the nasopharyngeal swab using the High Pure Viral RNA Kit (Rocha, Switzerland), the 5 μl extracted viral RNAs were amplified by quantitative reverse transcription polymerase chain reaction (qRT-PCR) using commercial kit from Genekey, China (GK-RP-114N V3). Samples with a Ct value ≤ 38.0 were considered putatively positive, while samples with Ct > 38.0 were considered negative. Viral burden was calculated based on a standard curve produced using serial 10-fold dilutions of SARS-CoV-2 RNA.

### Pseudovirus Neutralization Assay

SARS-CoV-2 pseudovirus neutralization assay was carried out on Huh-7 cell in 96-well microplate. Briefly, serum samples were diluted in three-fold dilutions with a beginning dilution of 1:20 and mixed with 50 μl /well of 2 × 10^4^ TCID_50_/ml pseudovirus. After incubating for 60 min at 37°C, 100 μl of 2 × 10^5^ /ml Huh-7 cell was added to each well. The mixtures were incubated for 20-28 h at 37°C with 5% CO_2_. Steady-Glo luciferase assay kit (cat# E2520) purchased from Promega (USA) was used to detect luciferase activity in cell lysis. The inhibition rate was calculated with the following formula. Neutralizing activity was calculated with inhibition rate according to Reed-Muench Method.

$$\begin{array}{l} Inhibition\;rate=\left(1-\frac{sample\;luciferase\;activity\;}{pseudovirus\;luciferase\;activity}\right)\times 100\% \end{array}$$

### Enzyme-Linked Immunosorbent Assay (ELISA)

IgA, IgG, and IgM against SARS-CoV-2 S1+S2, NP, RBD protein in serum were detected by indirect ELISA. 96 well EIA plates were coated with 250 ng/well of S1+S2, NP protein or 150 ng/well of RBD protein separately overnight at 4°C. The plates were blocked with 200 μl/well of 2% Bovine Serum Albumin (Sigma-Aldrich, USA) in 0.05% PBST (1×phosphate buffered saline supplemented with 0.05% Tween-20) overnight at 4°C. Serum samples were diluted in ten-fold dilutions with blocking buffer, 100 μl/well of samples diluted to 1:10^3^-1:10^6^ were then added to the blocked plates and incubated for 2 h at room temperature (RT). Plates were then washed three times with 0.05% PBST and added with HRP-labeled goat anti-human IgA, IgG, IgM (Abcam, UK) diluted to 1:20000 or HRP-labeled mouse anti-human IgG1, IgG2, IgG3, IgG4 (Southern Biotech, USA) diluted to 1:4000 in blocking buffer. After incubating for 1 h at RT, plates were washed six times and incubated with TMB substrate (Solarbio, China) for 25 min at RT. The reaction was stopped with ELISA stop solution (Solarbio, China), the OD_450_ was read on BioTek (Synergy HTX, USA). A positive control (serum sample FS B26 with strongly neutralization activity in micro-neutralization assay is kindly provided by Guangdong Provincial Center for Disease Control and Prevention) diluted in ten-fold dilutions was set on every ELISA plate to normalization all the detected values on different plates.

### Cytokine Measurements

Cytokine concentration was measured by using 48-plex Bio-Plex Pro Human Cytokine Assays (Bio-Rad, USA) according to the manufacturer's instructions. 1:3 diluted serum in sample dilution buffer were incubated with magnetic beads for 1 h, then washed 3 times with washing buffer and incubated the beads with detection buffer for 30 min. Streptavidin-PE was added and incubated with beads for 10 min after wash. Bio-Plex 200 (Bio-Rad, USA) was used to get the reads of cytokines.

### Statistics

All the continuous variables and categorical variables in this study were expressed as median (IQR) and number/sum (%), respectively. Kruskal-Wallis test was used to compare the differences among multiple data group. Mann-Whitney *U*-test was used to two-group continuous variables comparison. χ^2^ test or Fisher's exact test was used to analyze two-group categorical variables. The correlations between neutralization titers and different protein binding detected in ELISA or cytokines level were evaluated by Spearman correlation coefficient. A two-tailed *P* < 0.05 was considered statistically significant, (^*^P values of < 0.05, ^**^P values of ≤ 0.01, ^***^P values of ≤ 0.001, ^****^P ≤ 0.0001). Statistical analysis of clinical data was performed using SPSS Statistics version 25 software (IBM, Armonk, NY, USA). All the experimental data was analyzed in GraphPad Prism software, version 8.

## Results

### The Demographic Characteristics and Laboratory Findings of COVID-19 Patients

A total of 63 patients admitted to hospitals in Shenzhen from January to March with various clinical features of COVID-19 (14 with severe symptoms, 23 with moderate symptoms, 20 with mild symptoms, and 6 from asymptomatic cases) were enrolled in this study. All the patients were positive with SARS-CoV-2 nucleic acid testing of nasopharyngeal swab. The demographic and clinical features of these patients were described in detail in Supplementary Table 1. Similar with other studies (21, 23), we observed a higher median age correlated with the disease severity among the patients. Among the many chronic conditions reported in these COVID-19 patients, cardiovascular disease was likely a comorbidity factor to accelerate COVID-19 disease severity (*P* = 0.049). Common manifestations in our cohort included fever, cough, sputum production, diarrhea, myalgia, or fatigue. Other symptoms were less common. Notably, dyspnoea (*P* < 0.001), myalgia or fatigue (*P* = 0.001), high temperature (*P* = 0.006) were the most common clinical symptoms reported in severe cases compared to other patients in this study.

As listed in Table 1, in consistent with other studies (23), hematological analysis showed significant low median of lymphocyte count and the percentage of T cells in severe cases compared to patients with mild diseases. Furthermore, the severe patients had decreased levels of albumin but increased levels of alanine aminotransferase (ALT), procalcitonin, lactate dehydrogenase compared to the patients with the moderate and mild COVID-19. Platelet counts of severe and moderate cases were lower than other patients. C-reactive protein (CRP) concentration and erythrocyte sedimentation rate were also remarkedly increased with the disease severity. Collectively, these results suggest increased systemic inflammation and comprised T cell response are associated with the severity of COVID-19 patients.

**Table 1:** Laboratory findings in patients with SARS-CoV-2 infection.

	**Total (*n* = 63)**	**Severe (*n* = 14)**	**Moderate (*n* = 23)**	**Mild (*n* = 20)**	**Asymptomatic (*n* = 6)**	***P*-value**	***P*-value of comparison between groups**
Neutrophil count, ×10^9^ per L (*N* = 54)	2.76 (1.90-4.37)	3.13 (2.13-4.38)	2.57 (1.67-4.37)	2.94 (1.93-4.48)	NA	0.668	
Lymphocyte count, ×10^9^per L (*N* = 54)	1.36 (0.97-1.87)	0.90 (0.83-1.20)	1.28 (0.89-1.67)	1.95 (1.51-2.66)	NA	<0.001	<0.001^B^
							<0.001^C^
T lymphocyte,% (*N* = 47)	68.30 (61.10-72.50)	57.80 (48.40-69.60)	70.70 (66.00-76.50)	68.30 (64.80-77.10)	NA	0.020	0.008^A^
Helper T lymphocytes, % (*N* = 47)	37.80 (28.30-45.70)	32.90 (26.30-44.15)	40.00 (31.70-49.90)	36.20 (28.30-42.10)	NA	0.344	
Cytotoxic T lymphocyte, % (*N* = 47)	23.60 (17.00-31.50)	17.00 (13.80-25.75)	23.60 (17.00-36.00)	24.90 (22.20-29.40)	NA	0.163	
CD3^+^CD4^+^/CD3^+^CD8^+^ (*N* = 47)	1.57 (1.04-2.41)	2.03 (1.18-2.76)	1.77 (0.94-2.65)	1.38 (1.15-1.67)	NA	0.497	
Monocyte count, ×10^9^ per L (*N* = 54)	0.42 (0.31-0.54)	0.33 (0.25-0.49)	0.44 (0.33-0.53)	0.44 (0.40-0.60)	NA	0.207	
Platelet count, ×10^9^ per L (*N* = 54)	191 (146-269)	147 (118-196)	178 (127-268)	254 (187-312)	NA	0.002	0.476^A^
							0.002^B^
							0.056^C^
CRP, mg/L (*N* = 54)<8	6.00 (1.41-23.37)	29.30 (18.44-62.76)	6.45 (3.49-16.84)	1.37 (0.35-4.38)	NA	<0.001	<0.001^A^
							<0.001^B^
							0.001^C^
D-dimer, mg/L (*N* = 54) 0-0.5	0.39 (0.24-0.64)	0.45 (0.39-0.97)	0.28 (0.22-0.50)	0.31 (0.22-0.59)	NA	0.052	
Hemoglobin, g/L (*N* = 53)	130 (123-141)	132 (125-140)	124 (118-144)	130 (124-139)	NA	0.665	
Erythrocyte sedimentation rate, mm/h (*N* = 54)	29 (17–59)	61 (32–82)	29 (20–50)	16 (6–23)	NA	<0.001	0.013^A^
							<0.001^B^
							0.006^C^
Albumin, g/L (*N* = 55)	42.8 (40.6-45.9)	39.7 (38.5-42.1)	43.3 (40.6-45.9)	45.7 (42.1-48.1)	NA	<0.001	0.005^A^
							<0.001^B^
ALT, U/L (*N* = 55)	20.0 (13.0-28.0)	27.0 (26.1-41.4)	18.0 (13.0-24.0)	16.0 (11.0-21.4)	NA	0.001	0.002^A^
							<0.001^B^
AST, U/L (*N* = 55)	26.7 (20.0-37.1)	42.1 (24.5-51.1)	25.0 (19.0-31.9)	24.0 (18.0-37.1)	NA	0.065	
Total bilirubin, mmol/L (*N* = 55)	10.2 (6.8-13.0)	10.2 (8.5-13.1)	8.9 (6.8-13.0)	10.3 (6.1-15.7)	NA	0.859	
Potassium, mmol/L (*N* = 55)	3.80 (3.48-4.00)	3.68 (3.40-3.91)	3.80 (3.60-3.97)	3.81 (3.40-4.20)	NA	0.393	
Sodium, mmol/L (*N* = 55)	139.4 (136.8-141.0)	137.1 (135.5-140.5)	139.4 (136.8-140.6)	140.0 (138.0-141.5)	NA	0.108	
Procalcitonin, ng/mL (*N* = 55)	0.035 (0.024-0.056)	0.055 (0.049-0.116)	0.032 (0.024-0.048)	0.031 (0.020-0.050)	NA	0.002	<0.001^A^
							0.004^B^
Troponin I, pg/mL (*N* = 49)	0.012 (0.006-0.012)	0.012 (0.006-0.012)	0.012 (0.008-0.012)	0.012 (0.006-0.014)	NA	0.918	
Lactate dehydrogenase, U/L (*N* = 47)	209.0 (169.0-260.0)	248.0 (201.0-291.5)	200.5 (160.3-231.5)	195.0 (170.0-227.5)	NA	0.048	0.035^A^
							0.025^B^

*SARS-CoV-2, severe acute respiratory syndrome coronavirus 2; ALT, Alanine aminotransferase; AST, Aspartate aminotransferase; CRP, C-reactive protein; IQR, interquartile range. Data sets are shown as median (IQR). P values comparing the difference among four groups is from Kruskal-Wallis test. Differences comparing between two groups are from χ2 test, Fisher's exact test or Mann-Whitney U test. Missing values are shown as NA. A, B, C represent the P value of comparison between severe cases and moderate cases, severe cases and mild cases, moderate cases and mild cases, respectively*.

### SARS-CoV-2- Specific Antibody Responses in COVID-19 Patients

**Figure 1:**
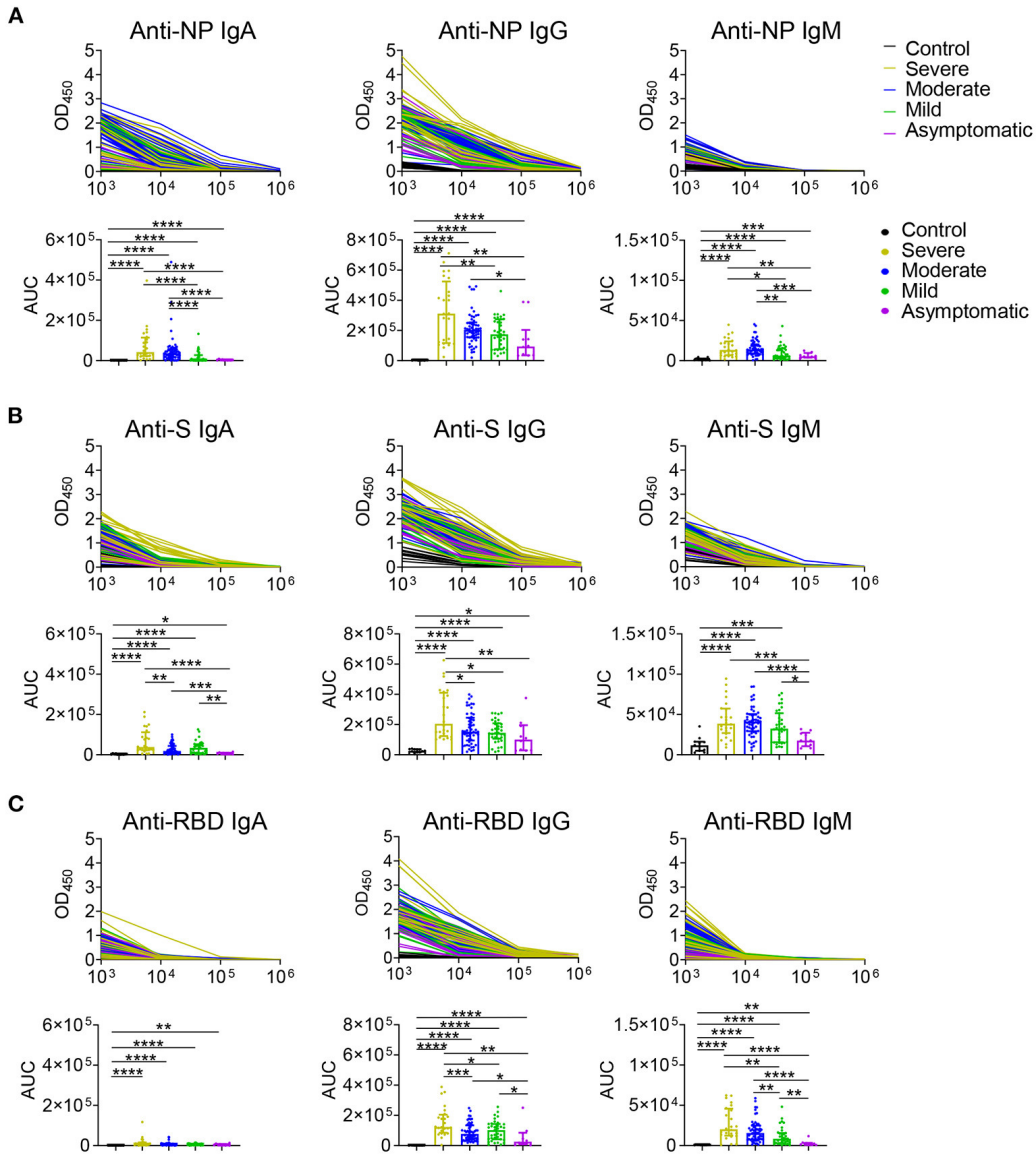
IgA, IgG, and IgM antibodies responses in COVID-19 ranging from asymptomatic to severe patients. Serum samples collected from COVID-19 patients were used for detecting IgA, IgG, and IgM levels to NP **(A)**, S **(B)**, and RBD **(C)** antigens of SARS-CoV-2 via ELISA. Antibody titers of the healthy controls (*n* = 11) and the severe (*n* = 24), moderate (*n* = 54), mild (*n* = 35), asymptomatic (*n* = 10) patients were shown in **(A–C)**. Mann-Whitney *U*-test was used to compare differences of medium values between groups, a two-tailed *P* value < 0.05 was considered to be statistically significant. ^*^P < 0.05 or ^**^P ≤ 0.01 or ^***^P ≤ 0.001 or ^****^P ≤ 0.0001 for the comparison between two groups.

**Figure 2:**
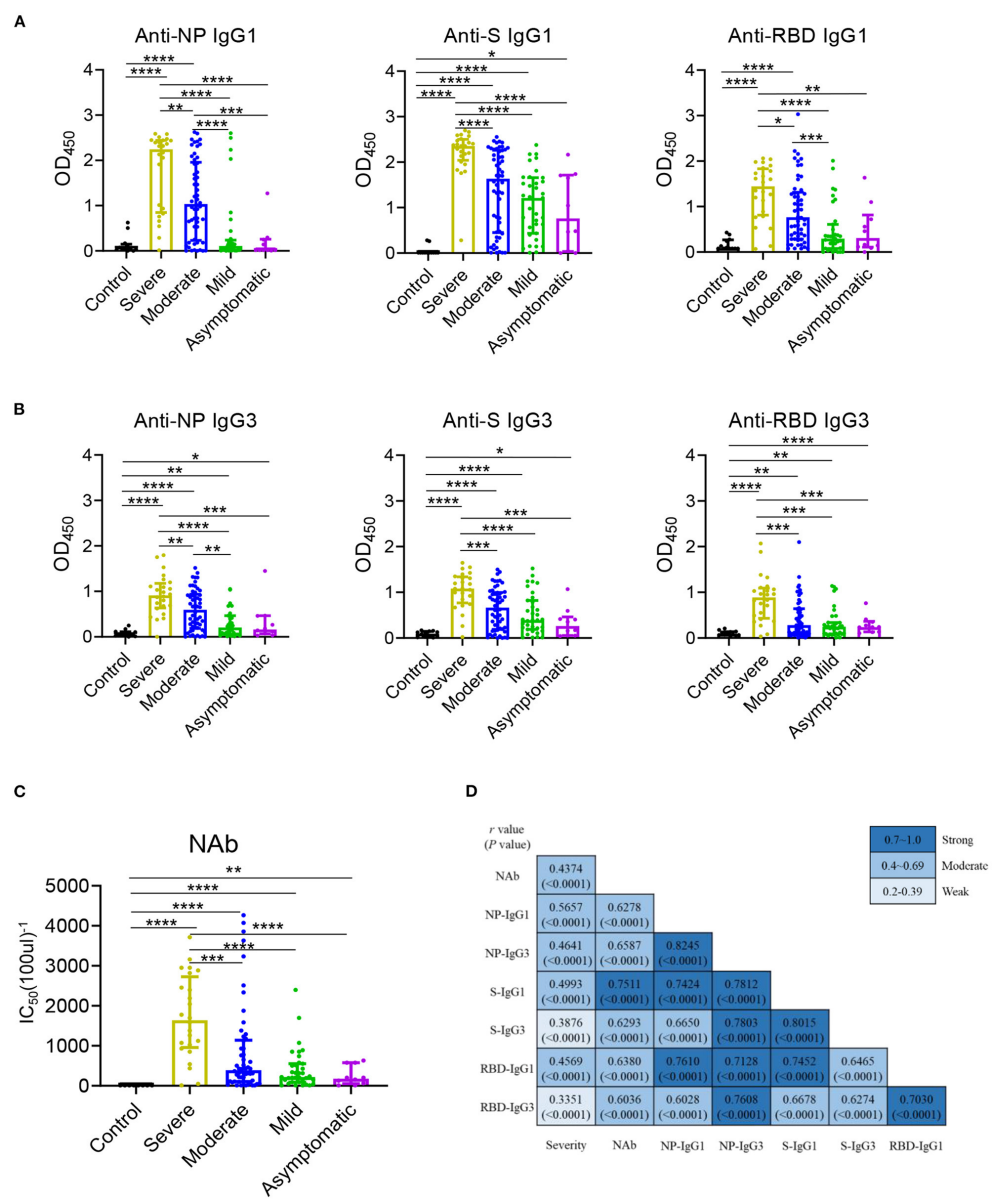
IgG1 and IgG3 were the main subclasses induced in COVID-19 patients and related with disease severity. IgG1 **(A)** and IgG3 **(B)** responses to NP, S, and RBD and neutralizing antibody **(C)** against SARS-CoV-2 pseudo-virus with luciferase reporter gene in the healthy controls (*n* = 11) and the severe (*n* = 24), moderate (*n* = 54), mild (*n* = 35), asymptomatic (*n* = 10) patients were detected. Differences of medium values between groups were analyzed by Mann-Whitney *U*-test. Significant correlations among Severity, NAb and IgG subclasses **(D)** including anti-NP IgG1, IgG3, anti-S IgG1, IgG3, anti-RBD IgG1, IgG3 were shown. Spearman correlation coefficient was calculated. A two-tailed *P* value < 0.05 was considered to be statistically significant. ^*^P < 0.05 or ^**^P ≤ 0.01 or ^***^P ≤ 0.001 or ^****^P ≤ 0.0001 for the comparison between two groups.

The serum levels of immunoglobulin (Ig) isotypes including IgA, IgG, and IgM responses and IgG subclasses against SARS-CoV-2 nucleocapsid protein (NP), spike (S), and receptor binding domain (RBD) antigens were measured by ELISA. The enrolled samples were collected between 0 and 51 days after disease onset and serum of healthy people were collected as controls. Utilizing individual serum with serial dilutions, the area under the curve (AUC) was determined to calculate antibody titer. As shown in Figures 1A–C, the severe patients had higher Ig isotypes titers compared to the asymptotic patients except RBD- specific IgA, and stronger Ig isotypes than the mild patients except S- specific IgA, IgM or RBD specific IgA, and higher AUCs of S- specific IgA, IgG, and RBD- specific IgG than the moderate patients. Besides, NP-, S-, RBD- specific IgG1 and IgG3 increased with the disease severity among COVID-19 patients (Figures 2A,B); whereas IgG2 and IgG4 subclasses were barely detectable (Supplementary Figures 1A,B).

The NAb activities were detected in COVID-19 patients using a well-established pseudovirus with luciferase reporter assay (26, 27). While the NAb titer was highest in patients with severe diseases, no significant differences were found among other COVID-19 patients (Figure 2C). A modest to strong correlations were noted between the NAb activities to the levels of antigen specific IgG1 and IgG3 (Figure 2D) other than IgG2 and IgG4 (Supplementary Figure 1C) while mild to moderate correlations were observed between the NAb and Ig isotypes (Supplementary Figure 2). Besides, all the IgG1/ IgG3 against NP, S and RBD exhibited a modest to strong correlations with each other (Figure 2D). Further, the correlations between disease severity and Ig isotypes, IgG subclasses were compared, NAb, NP- specific IgA, RBD- specific IgM (Supplementary Figure 2) and NP, S, RBD- specific IgG1 and NP- specific IgG3 were modestly associated with the disease severity (Figure 2D).

**Figure 3:**
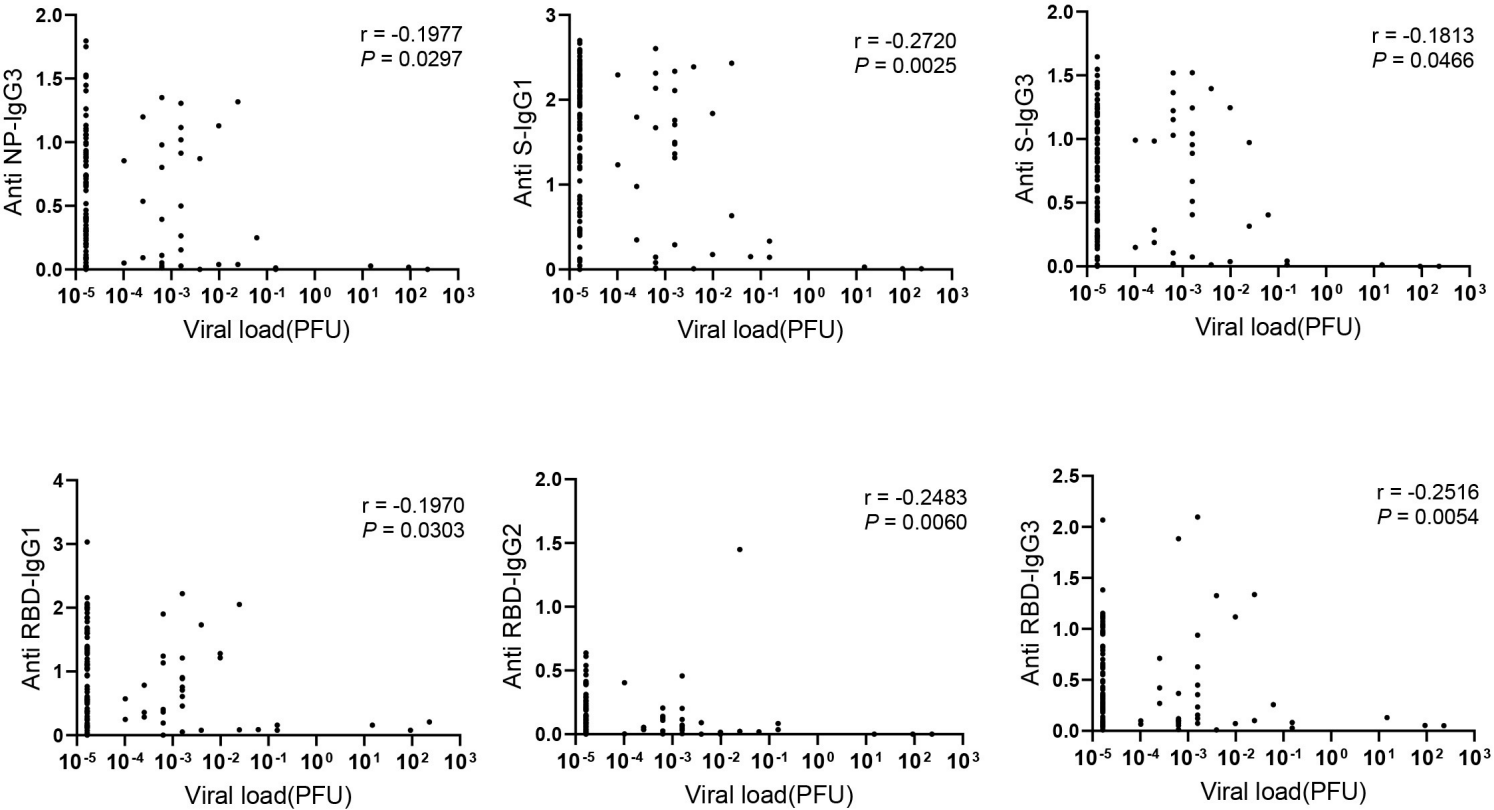
The negative correlation between IgG subclasses and viral load. Correlations between antibody levels in 121 COVID-19 serum samples and corresponding viral load detected in the nasopharyngeal swab were applied. Spearman correlation coefficient was calculated. A two-tailed *P* value < 0.05 was considered to be statistically significant.

To figure out the association of viral load in COVID-19 patients with antibody response, RNA level of nasopharyngeal swabs was measured and calculated as viral load due to the limited detection of viral RNA in serum. Interestingly, IgG subclasses in serum including NP- specific IgG3, S- specific IgG1, IgG3 and RBD- specific IgG1, IgG2, IgG3 showed weakly negative correlation with viral load in nasopharyngeal swab (Figure 3). However, we did not find the significant correlation between the viral load and the total Ig isotypes (data not shown).

### Antibody Associated Cytokine Productions in COVID-19 Patients

**Figure 4:**
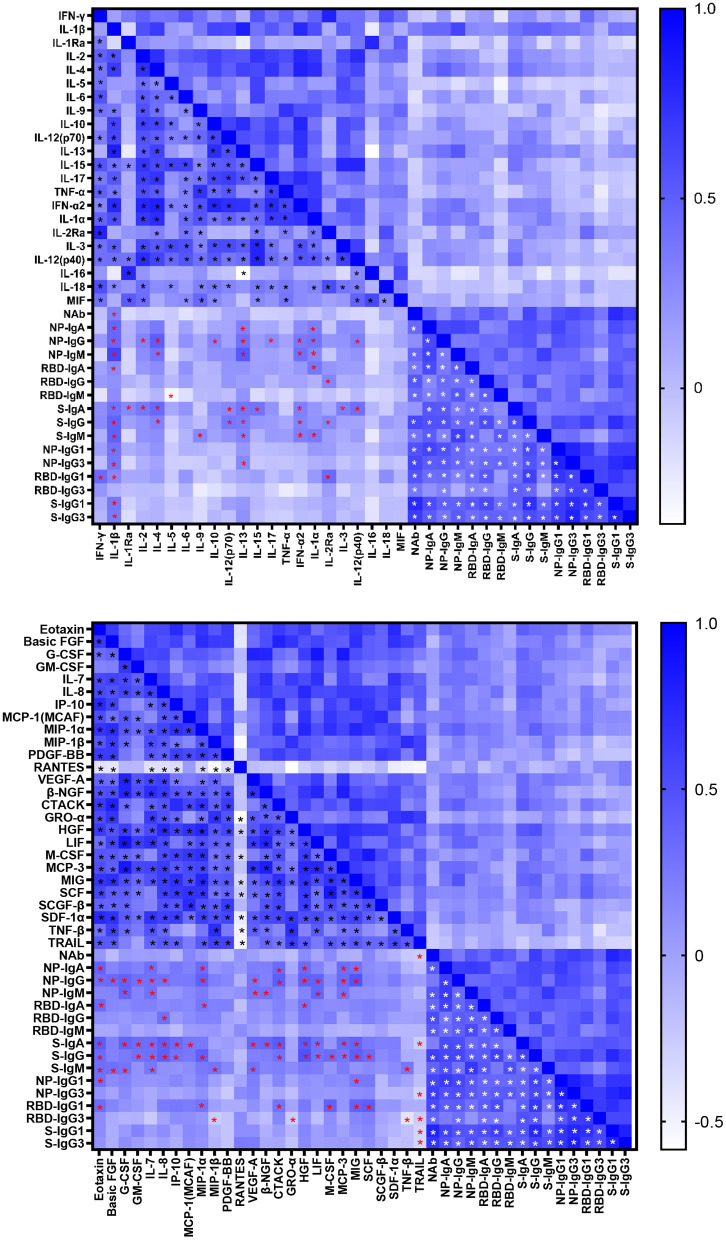
Correlations between cytokine and antibody levels in COVID-19 patients. Spearman correlation coefficient between cytokines and neutralizing antibody, IgA, IgG, IgM, IgG1, and IgG3 responses to NP, RBD and S in COVID-19 patients were evaluated. A two-tailed *P* value < 0.05 was considered to be statistically significant and marked with black, red and white asterisks which represents correlation between cytokines, cytokine and antibody, antibodies respectively.

Cytokines induced in serum collected between 0 and 51 days after disease onset were analyzed by Bio-plex assays. As shown in Supplementary Figure 3, various inflammatory cytokines, chemokines and growth factors were induced in COVID-19 patients. The correlations between SARS-CoV-2 specific antibodies and cytokines were shown in Figure 4. The NAb was correlated with interleukin (IL)-1β positively while with TRAIL negatively. Most cytokines had a positive association with Ig isotypes, of which IL-1β was positively correlated with most of antigen specific Ig isotypes and IgG subclasses except IgG, IgM, IgG3 against RBD. Interestingly, we had found a significant negative association between IL-5 and RBD- specific IgM. As the main subclasses of IgG, IgG1, or IgG3 had positive association with IFN-γ, IL-13, IL-2Rα, Eotaxin, MIP-1α, MIP-1β, CTACK, GRO-α, M-CSF, MIG, SCF, SCGF-β, and negative correlation with TNF-β and TRAIL. Of note, the inflammatory cytokines IL-6 and tumor necrosis factor (TNF)-α were not significantly related to the antibody response.

### IgG1 and IgG3 Subclasses Kinetics During the Course of Disease Development

**Figure 5:**
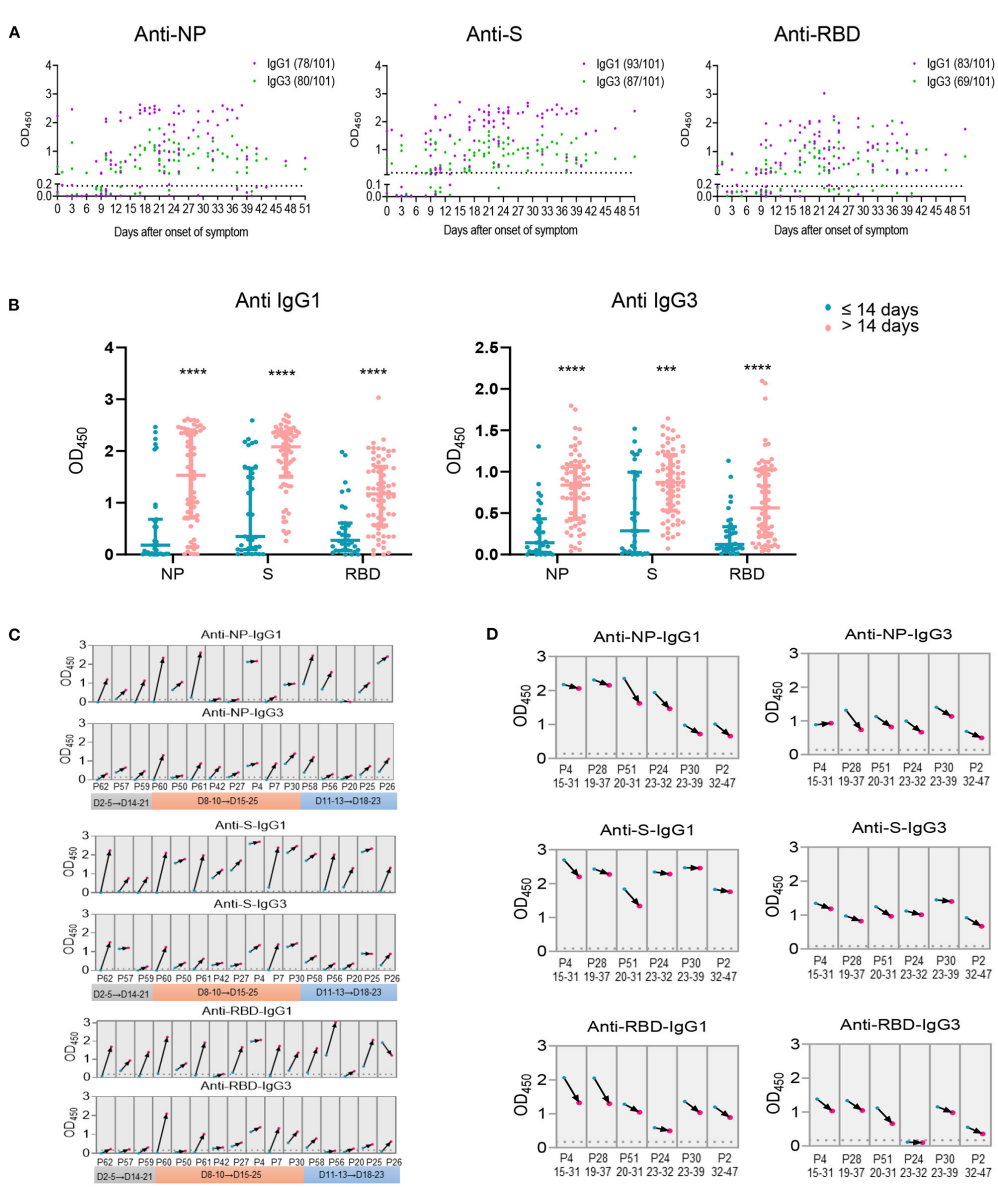
Antigen specific IgG subclasses production over days after onset of disease. Antigen specific IgG subclasses **(A)** induced in 101 serum samples collected from COVID-19 patients at different time points of symptoms onset were detected by ELISA. The levels of IgG1 and IgG3 against NP, S and RBD **(B)** within or beyond 14 days after onset of disease were summarized. Seroconversion of IgG1 and IgG3 against NP, S and RBD among 16 COVID-19 patients during the acute phase (the first 4 weeks since onset of disease) **(C)** and 6 patients during the recovery phase (the second 4 weeks since onset of disease) **(D)** was analyzed. The first sample (blue) and follow-up sample (red) are connected by black arrows. The time intervals between the first and follow-up samples are provided on the x axis. The cut off value was indicated by the broken line. ^***^P ≤ 0.001 or ^****^P ≤ 0.0001 for the comparison between two groups.

As we have found the significant negative association for IgG1 and IgG3 with viral load, the kinetics of IgG1 and IgG3 were analyzed utilizing serum samples within 51 days after disease onset. Unlike the total Ig isotypes were observed in most patients within the first week after onset of disease (Supplementary Figure 4A), IgG1 and IgG3 against NP, S or RBD of several cases were barely detectable within the first 2 weeks after symptoms onset (Figure 5A). Comparable occurrence of NP-, S- specific IgG1 and IgG3 were observed, whereas RBD- specific IgG3 showed a lower frequency than IgG1 (Figure 5A). Thus, compared with samples collected within 14 days after onset of disease, levels of IgG1 and IgG3 were markedly elevated in serum collected after 14 days of disease onset (Figure 5B). These data indicated an overall increasing IgG1 and IgG3 response after 14 days of disease onset compared the first two weeks, consisting with the NAb and total IgG isotypes in increase after two weeks of disease onset (Supplementary Figure 4B).

Furthermore, antibody responses of 11 individuals with two collections from each patient at different times of disease onset were analyzed. We observed overall increasing IgG1 and IgG3 trends in the specimens collected during the 3rd or 4th weeks compared with those collected in the first 2 weeks after onset of disease (Figure 5C). Besides, two harvesting with at least 10 days interval from 5 patients were collected 14 days after disease onset, an overall decreased antibody response trend was observed for the second serum collection than the first one (Figure 5D), suggesting a declined IgG subclasses response 4 weeks post disease onset.

### Age, Comorbidities, and Biological Sex-Associated Differential Immune Responses in COVID-19 Patients

**Figure 6:**
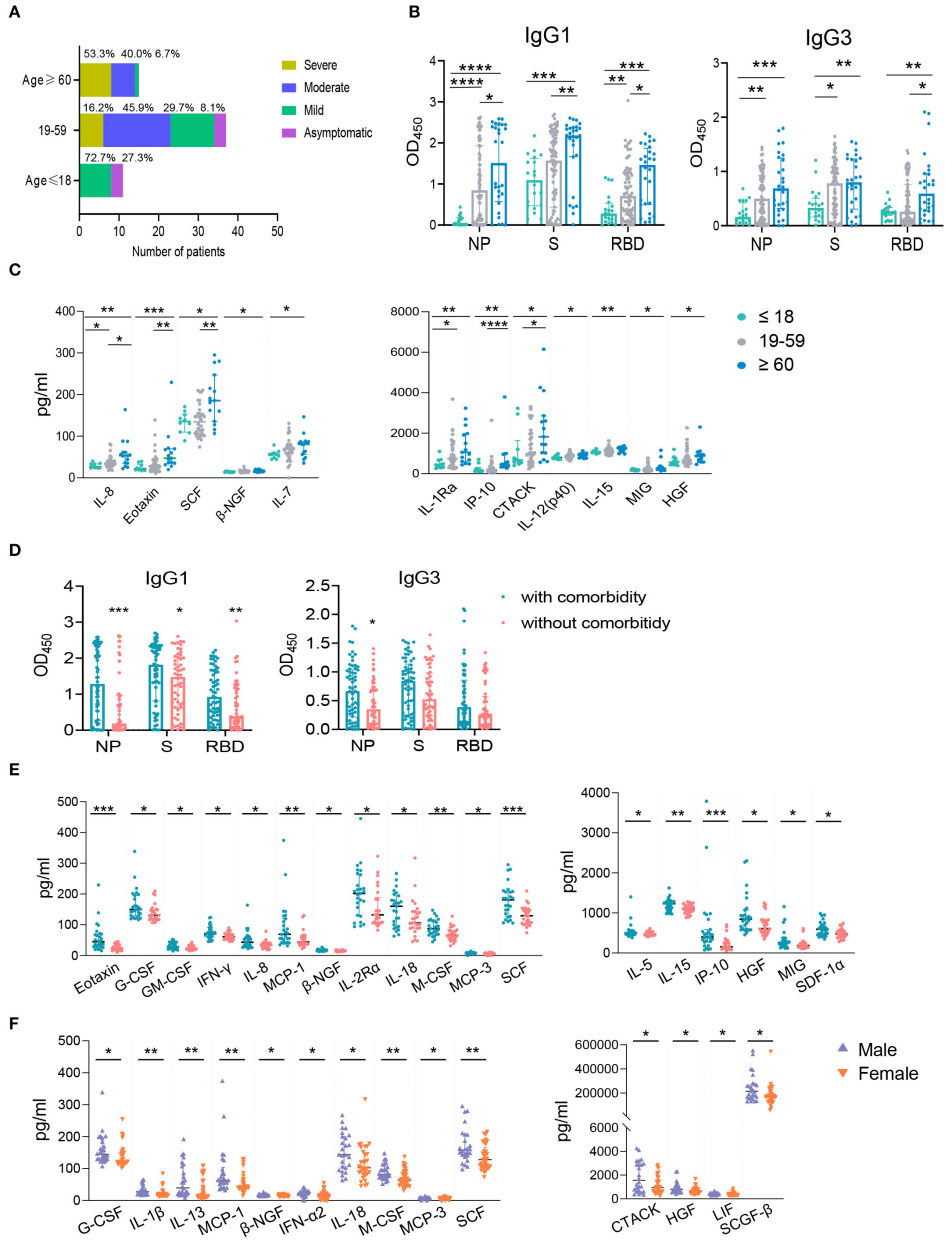
The influence of age, comorbidity, and biological sex on IgG subtypes or cytokines. The samples were classified into the young (≤ 18 years old, *n* = 20), the intermediate (19–59 years old, *n* = 75) and the old (≥ 60 years old, *n* = 28) groups according to the patients' age. The percentages of COVID-19 patients with different clinical outcomes were shown **(A)**. IgG subtypes against NP, S, and RBD between different age groups **(B)** and the groups with and without comorbidity **(D)** were evaluated. The influence of age **(C)**, comorbidity **(E)**, and biological sex **(F)** on cytokines were applied. Mann-Whitney *U-*test was used to compare differences of medium values between groups, a two-tailed *P* value < 0.05 was considered to be statistically significant. ^*^P < 0.05 or ^**^P ≤ 0.01 or ^***^P ≤ 0.001 or ^****^P ≤ 0.0001 for the comparison between two groups.

Previous studies have demonstrated the advancing age, comorbidities including hypertension, diabetes, cardiovascular disease, chronic liver disease, and male are risk factors of COVID-19 and drive higher antibody response (21, 23, 28–33). To further explore the effect of these risk factors on host immune response, we first analyzed the clinical features and immune response in the following age groups: young (≤ 18 years), intermediate (19–59 years), old (≥ 60 years). As expected, old patients were associated with comorbidities, such as diabetes, hypertension, and cardiovascular disease. No comorbidity was found in the young age group (Supplementary Table 2). In consistent with this, 53.3% of old patients developed severe disease; while most young patients showed either mild (72.7%) or no symptom (27.3%) (Figure 6A). The intermediate age group mostly included moderate (45.9%) and mild patients (29.7%) (Figure 6A). Although higher NAb and NP-, S-, RBD- specific IgA and NP-, RBD- specific IgM were higher in the old population compare to the young group, no significant difference was found between these two groups for total IgG (Supplementary Figure 5A). However, IgG1 and IgG3 responses against NP, S or RBD were much lower in the young group than the old group (Figure 6B). Besides, compared with young patients, the levels of a number of cytokines including IL-8, Eotaxin, SCF, β-NGF, IL-7, IL-1Ra, IP-10, CTACK, IL-12 (p40), IL-15, MIG, and HGF were higher in the old patients (Figure 6C), many of which were chemokines. Helper T cells are important for humoral response development, we noted the increased level of helper T cells in the old patients compared to the young patients (Table 2), which were consistent with results of antibody response. Further, the overall lymphocyte count was decreased with age. Increased CD3^+^CD4^+^/CD3^+^CD8^+^ T cells but markedly reduced percentage of cytotoxic T cells was observed in the old patients than other patients (Table 2). Thus, the advancing age is a risk factor which enhanced chemokines and SARS-CoV-2 specific antibody responses and dampened functional T cells.

**Table 2:** Age- related laboratory findings patients with SARS-CoV-2 infection.

	**Age ≤ 18 (*N* = 11)**	**19 ≤ Age ≤ 59 (*N* = 37)**	**Age ≥ 60 (*N* = 15)**	***P*-value**	***P*-value of comparison between groups**
Neutrophil count, ×10^9^ per L	1.94 (0.95-3.05)	2.74 (1.97-4.33)	3.57 (2.20-4.80)	0.113	
Lymphocyte count, ×10^9^ per L	3.25 (2.06-5.37)	1.35 (1.05-1.78)	0.99 (0.87-1.48)	<0.001	0.003^A^
					<0.001^B^
T lymphocyte,%	67.2 (62.5-68.6)	70.7 (63.7-78.6)	66.0 (55.7-70.2)	0.036	0.021^C^
Helper T lymphocytes, %	31.70 (25.70-36.00)	37.70 (30.00-42.15)	45.70 (30.90-49.90)	0.055	
Cytotoxic T lymphocyte,%	23.0 (22.2-24.9)	29.8 (19.5-35.9)	17.0 (11.6-23.0)	<0.001	0.001^C^
CD3^+^CD4^+^/CD3^+^CD8^+^	1.41 (1.08-1.57)	1.24 (0.89-2.13)	2.65 (1.91-3.30)	<0.001	0.001^C^
Monocyte count, ×10^9^ per L	0.41 (0.29-0.70)	0.45 (0.34-0.54)	0.37 (0.26-0.52)	0.493	
Platelet count, ×10^9^ per L	218.0 (176.5-277.5)	204.5 (148.5-271.5)	164.5 (129.3-229.5)	0.294	
CRP, mg/L	3.03 (0.69-5.44)	5.50 (1.37-16.09)	25.43 (6.88-69.54)	0.011	0.024^B^
					0.033^C^
D-dimer, mg/L	0.40 (0.30-0.64)	0.30 (0.22-0.53)	0.45(0.28-0.78)	0.239	
Hemoglobin, g/L	128.5 (124.3-139.8)	132.0 (123.0-143.0)	126.0 (114.8-135.8)	0.377	
erythrocyte sedimentation rate, mm/h	11.50 (5.25-17.25)	28.00 (17.00-40.00)	60.00 (33.00-83.00)	<0.001	0.046^A^
					<0.001^B^
					0.017^C^
Albumin, g/L	45.80 (42.85-48.85)	42.55 (40.45-46.28)	41.60 (38.60-43.80)	0.052	
ALT, U/L	16.05 (10.48-29.18)	17.50 (11.25-26.50)	26.20 (18.00-33.30)	0.099	
AST, U/L	32.55 (24.98-40.25)	22.00 (18.25-28.00)	35.00 (26.00-45.30)	0.005	0.014^C^
Total bilirubin, mmol/L	7.00 (5.50-10.85)	10.70 (7.93-15.53)	9.60 (6.80-14.50)	0.108	
Potassium, mmol/L	4.10 (3.83-4.68)	3.73 (3.44-3.96)	3.76 (3.50-3.90)	0.024	0.028^A^
					0.044^B^
Sodium, mmol/L	139.5 (138.0-140.9)	139.2 (136.9-140.6)	140.0 (136.2-141.1)	0.904	
Procalcitonin, ng/mL	0.056 (0.035-0.168)	0.032 (0.020-0.049)	0.049 (0.029-0.095)	0.026	0.012^A^
Troponin I, pg/mL	0.012 (0.008-0.012)	0.010 (0.006-0.012)	0.012 (0.009-0.013)	0.095	
Lactate dehydrogenase, U/L	231.0 (215.8-275.8)	186.0 (160.5-213.5)	254.0 (198.0-323.5)	0.006	0.023^A^
					0.004^C^

*SARS-CoV-2, severe acute respiratory syndrome coronavirus 2; ALT, Alanine aminotransferase; AST, Aspartate aminotransferase; CRP, C-reactive protein; IQR, interquartile range. Data sets are shown as median (IQR). P values comparing the difference among three groups is from Kruskal-Wallis test. Differences comparing between two groups are from χ2 test, Fisher's exact test or Mann-Whitney U test. A, B, C mean the P value of comparison between group Age ≤ 18 and 19 ≤ Age ≤ 59, Age ≤ 18 and Age ≥ 60, 19 ≤ Age ≤ 59 and Age ≥ 60, respectively*.

Except higher NAb and NP- specific IgA and IgM (Supplementary Figure 5B), NP-, S-, RBD- specific IgG1 and NP- specific IgG3 subclass responses were higher in COVID-19 patients with comorbidity compared to patients without comorbidities (Figure 6D). Patients with comorbidity also had elevated levels of cytokines including Eotaxin, G-CSF, GM-CSF, IFNγ, IL-8, MCP-1, β-NGF, IL-2Rα, IL-18, M-CSF, MCP-3, SCF, IL-5, IL-15, IP-10, HGF, MIG, and SDF1α (Figure 6E). These results indicated that comorbidity is associated with higher IgG subclasses and multiple cytokines. However, our data showed that biological sex had no significant effect on IgG subclasses (data not shown) although male patients had a higher NAb and S- specific IgG responses than female patients (Supplementary Figure 5C). We also found male patients had increased levels of cytokines including G-CSF, IL-1β, IL-13, MCP-1, β-NGF, IFN-α2, IL-18, M-CSF, MCP-3, SCF, CTACK, HGF, LIF, SCGF-β than females (Figure 6F). Collectively, advancing age and comorbidities are associated higher IgG subclasses and multiple cytokines, while biological sex had no effect on IgG1 and IgG3.

## Discussion

Although antibody and cytokine responses have been recently reported in COVID-19 patients (23, 34, 35), their associations with viral pathogenesis and disease development are not clearly understood. In this study, we fully characterized the antibody and cytokine responses in COVID-19 patients with various clinical manifestations.

Due to the polyclonal nature, the antibodies display multiple function and features, thus the portion of antibody is important to control viral infection. During the outbreak of SARS-CoV-2, the total Ig isotypes against NP, S and RBD were well studied (36), however, the kinetics of IgG1 and IgG3 in COVID-19 patients was not fully described. In line with the findings in two recent reports (37, 38), our study indicated that SARS-CoV-2 specific IgG1 and IgG3 were the dominant subclasses of IgG; while IgG2 and IgG4 were barely detected in COVID-19 patients. Chen et al. also showed higher levels of RBD-specific IgG1 and IgG3 in severe COVID-19 patients compared to non-severe patients. Here, we further extended the comparison to patients with various disease severity, including moderate, mild and asymptomatic symptoms, and performed the correlation analysis between IgG subclasses and the disease severity. We have found that disease severity was related to multiple antigen-specific antibodies including NP- specific IgG1 and IgG3. One recent study reported that severe COVID-19 patients showed a trend of higher levels of IgG1 with afucosylated Fc glycans, which would enhance the interactions with the activating Fc receptor and induce inflammatory cytokines in monocytes. This is likely to be a mechanism to support the correlation between IgG subclasses (IgG1) to the disease severity of COVID-19 (39).

The relationship between antibodies and viral load is another important concern. Our studies demonstrated that NAb, IgA, and total levels of IgG and IgM were not correlated with viral load in COVID-19 patients, while a weakly inverse correlation between IgG subclasses and viral load was observed. Our findings are consistent with two prior studies, which also reported no correlation between persistent SARS-CoV-2 RNA and NAb titers (24, 40). Slim Fourati et al. reported a lower early antibody responses were related to higher viral load in nasopharyngeal swabs in the severely ill COVID-19 patients (41), though the study may be limited by its smaller sample size. Considering the negative correlation between IgG subclasses with viral load, the role of IgG subclasses especially IgG1 and IgG3 in COVID-19 patients should be valued.

Recent studies have reported that the deceased COVID-19 patients have more NP- specific humoral responses while the convalescents individual's antibody response is S-centric (42), suggesting the antigen specific antibodies influence the immunity effectiveness and disease development. Interestingly, although RBD specific antibody has been shown to be the dominant in COVID-19 patients, here, we noticed that P24 and several other patient samples showed high binding activity with S protein, but lower or almost no binding with the RBD domain. It's likely that these patients produced antibodies against other domains of S protein, such as the S2 domain (36).

The risk factors including advancing age, and comorbidities have more influence on IgG subclasses compared on the total IgG while biological sex mainly affect the total IgG, suggesting the IgG subclasses are affected by different risk factors. In the present study, the advancing age is not just associated with higher antibodies response and dampened T cell function, but also with hyperinflammation mainly induced by chemokines. Actually, the advancing age is considered as the most common comorbidity (43), thus the similar chemokine profiles is shown between the old and individuals with comorbidities. Chemokines such as IL-8 and IP-10 are higher in the old cases or patients with comorbidities, indicating that the common effect for advancing age and comorbidity on cytokines response in COVID-19 patients. Several studies have demonstrated the sex bias for male COVID-19 patients enhancing the disease severity (32, 44), and IL-8, IL-18, CCL5 are the cytokines associated with sex bias (45), our cohort suggests multiple cytokines including IL-1β, IFN-α2, MCP-1, and MCP-3 are also involved in sex bias of COVID-19 patients. Overall, the risk factors such as age and other comorbidities should be considered when analyzing the correlation between antibody response and the disease severity in future study with a larger group size of COVID-19 patients.

Though the antibody and cytokine profiles have been described in detail at the early stage of COVID-19 outbreak (23), the correlation between antibody and cytokines in COVID-19 were largely unknown. The matrix analysis indicates IL-1β is an essential factor related with NAb, Ig isotypes and even IgG subclasses. As the inflammatory cytokines, IL-1β is upregulated in COVID-19 patients (46, 47) and is secreted through the SARS-CoV-2-induced necroptosis pathway (48). The blocking of IL-1 signaling has been used to treat respiratory failure in COVID-19 (49). Regarding the significant association for IL-1β with antibody response, the use of IL-1β agonists to treat COVID-19 patients needs to be carefully evaluated.

Although this study showed several increased cytokines in the severe COVID-19 patients compared with mild, moderate and asymptomatic patients, we did not note differences on cytokine levels between mild and asymptomatic groups, which is different from the results of another study (23). One possibility is the number of asymptomatic patients enrolled in this study was relatively small as the asymptomatic population was not screened widely during collection of samples. In addition, we did not collect samples for multiple time points from the same patients and lost the follow up tracking after out of hospital.

Taken together, we have analyzed the antibody and cytokine responses in COVID-19 ranging from asymptomatic to severe patients and evaluated the effects of multiple risk factors, including comorbidities, male sex and advancing age on host immune response of COVID-19 patients. Although further studies with large cohorts are needed to demonstrate the accurate role of severity related higher antibody response in COVID-19 patients, our results showed NP-, S-, RBD- specific IgA, IgG, IgM are not associated with SARS-CoV-2 viral load, indicating there is no obvious correlation between antibody response and viral antigen detected in nasopharyngeal swabs. These data may provide novel insights to guide deployment of safe and effective immunomodulatory therapeutics.

## Data Availability Statement

The raw data supporting the conclusions of this article will be made available by the authors, without undue reservation.

## Ethics Statement

The studies involving human participants were reviewed and approved by The biomedical research ethics committee, the public health school (Shenzhen) of Sun Yat-sen University. Written informed consent to participate in this study was provided by the participants' legal guardian/next of kin.

## Author Contributions

YS, SF, and TW conceived and designed the study. HL and TJ performed experiments, analyzed data and wrote the manuscript. JC, SZ, ZQ, SW, and XL contributed to the data collection, data analysis and data interpretation. YL, XW, WW, RZ, XZ, and TF had roles in specimen collection and data collection. RD and YZ provided the pseudovirus. Y-QC and CS reviewed and approved the final version of the manuscript. All authors contributed to the article and approved the submitted version.

## Conflict of Interest

The authors declare that the research was conducted in the absence of any commercial or financial relationships that could be construed as a potential conflict of interest.
