# Supplementary material for: The Characterization of Disease Severity Associated IgG Subclasses Response in COVID-19 Patients
Source: Front Immunol. 2021 Mar 4;12:632814. doi: 10.3389/fimmu.2021.632814 (PMC7982848; doi:10.3389/fimmu.2021.632814)
Supplement: Supplementary file 1 [file Table_1.DOCX]

|  | **Total**  **(n=63)** | **Severe**  **(n=14)** | **Moderate**  **(n=23)** | **Mild**  **(n=20)** | **Asymptomatic**  **(n=6)** | ***P*-value** | ***P*-value of group comparison** |
| --- | --- | --- | --- | --- | --- | --- | --- |
| **Age**(N=63) | 45.00 (28.00-59.00) | 62.50 (46.75-65.25) | 50.00 (37.00-60.00) | 27.50 (7.50-38.25) | 24.50 (6.25-52.75) | <0.001 | 0.017^A^ |
|  |  |  |  |  |  |  | <0.001^B^ |
|  |  |  |  |  |  |  | 0.006^C^ |
|  |  |  |  |  |  |  | <0.001^D^ |
|  |  |  |  |  |  |  | 0.037^E^ |
| **Sex**(N=63) |  |  |  |  |  | 0.527 |  |
| Male, n/N(%) | 21/63 (33.3%) | 6/14 (42.9%) | 9/23 (39.1%) | 5/20 (25.0%) | 1/6 (16.7%) |  |  |
| Female, n/N(%) | 42/63 (66.7%) | 8/14 (57.1%) | 14/23 (60.9%) | 15/20 (75.0%) | 5/6 (83.3%) |  |  |
| **Baseline comorbidity** |  |  |  |  |  |  |  |
| Diabetes, n/N(%) | 7/56 (12.5%) | 2/13 (15.4%) | 4/23 (17.4%) | 1/20 (5.0%) | NA | 0.514 |  |
| Hypertension,n/N(%) | 8/56 (14.3%) | 4/13 (30.8%) | 3/23 (13.0%) | 1/20 (5.0%) | NA | 0.123 |  |
| Cardiovascular disease, n/N(%) | 4/56 (7.1%) | 3/13 (23.1%) | 1/23 (4.3%) | 0/20 (0.0%) | NA | 0.049 |  |
| Chronic liver disease, n/N(%) | 2/56 (3.6%) | 1/13 (7.7%) | 1/23 (4.3%) | 0/20 (0.0%) | NA | 0.701 |  |
| Operation history, n/N(%) | 16/56 (28.6%) | 4/13 (30.8%) | 8/23 (34.8%) | 4/20 (20.0%) | NA | 0.563 |  |
| **Signs and symptoms** |  |  |  |  |  |  |  |
| Fever, n/N(%) | 43/54 (79.6%) | 13/13 (100.0%) | 17/23 (73.9%) | 13/18 (72.2%) | NA | 0.117 |  |
| Cough, n/N(%) | 43/54 (79.6%) | 13/13 (100.0%) | 18/23 (78.3%) | 12/18 (66.7%) | NA | 0.072 |  |
| Pharyngalgia,n/N(%) | 7/54 (13.0%) | 0/13 (0.0%) | 4/23 (17.4%) | 3/18 (16.7%) | NA | 0.343 |  |
| Sputum production, n/N(%) | 30/54 (55.6%) | 10/13 (76.9%) | 13/23 (56.5%) | 7/18 (38.9%) | NA | 0.107 |  |
| Haemoptysis,n/N(%) | 1/54 (1.9%) | 0/13 (0.0%) | 1/23 (4.3%) | 0/18 (0.0%) | NA | 1.000 |  |
| Dyspnoea, n/N(%) | 9/54 (16.7%) | 8/13 (61.5%) | 1/23 (4.3%) | 0/18 (0.0%) | NA | <0.001 | <0.001^A^ |
|  |  |  |  |  |  |  | <0.001^B^ |
| Thoracalgia, n/N(%) | 4/54 (7.4%) | 2/13 (15.4%) | 2/23 (8.7%) | 0/18 (0.0%) | NA | 0.270 |  |
| Myalgia or fatigue, n/N(%) | 19/54 (35.2%) | 10/13 (76.9%) | 6/23 (26.1%) | 3/18 (16.7%) | NA | 0.001 | 0.004^A^ |
|  |  |  |  |  |  |  | 0.001^B^ |
| Headache, n/N(%) | 9/54 (16.7%) | 1/13 (7.7%) | 7/23 (30.4%) | 1/18 (5.6%) | NA | 0.076 |  |
| Diarrhoea, n/N(%) | 18/54 (33.3%) | 7/13 (53.8%) | 4/23 (17.4%) | 7/18 (38.9%) | NA | 0.072 |  |
| Highest temperature,℃  (N=52) | 37.90  (37.30-38.95) | 38.90  (38.25-39.50) | 37.95  (37.30-38.58) | 37.45  (37.03-37.93) | NA | 0.006 | 0.018^A^ |
|  |  |  |  |  |  |  | 0.002^B^ |
| Systolic pressure,mm Hg  (N=50) | 124.00  (114.00-136.50) | 128.00  (122.00-139.00) | 124.00  (110.00-138.00) | 117.50  (113.00-133.75) | NA | 0.270 |  |

**Supplementary table 1. Demographics and baseline characteristics of patients with SARS-CoV-2 infection.**

**Abbreviations:** SARS-CoV-2, severe acute respiratory syndrome coronavirus 2; IQR, interquartile range; Data are median (IQR) or n/N (%), where N is the total number of patients with available data. *P* values comparing the difference among four groups are from Kruskal-Wallis test. Differences comparing between two groups are from χ^2^ test, Fisher’s exact test or Mann-Whitney U test. Missing values are shown as NA. A, B, C, D, E represent the *P* value of comparison between severe cases and moderate cases, severe cases and mild cases, severe cases and asymptomatic cases, moderate cases and mild cases, moderate cases and asymptomatic cases, respectively.
