# Supplementary material for: The Characterization of Disease Severity Associated IgG Subclasses Response in COVID-19 Patients
Source: Front Immunol. 2021 Mar 4;12:632814. doi: 10.3389/fimmu.2021.632814 (PMC7982848; doi:10.3389/fimmu.2021.632814)
Supplement: Supplementary file 2 [file Table_2.DOCX]

|  | **Age ≤ 18**  **(n=11)** | **19 ≤ Age ≤ 59**  **(n=37)** | **Age ≥ 60**  **(n=15)** | ***P*** | ***P*-value of group comparison** |
| --- | --- | --- | --- | --- | --- |
| **Sex(**N=63**)** |  |  |  | 1.000 |  |
| Male,n/N(%) | 4/11 (36.4%) | 12/37 (32.4%) | 5/15 (33.3%) |  |  |
| Female,n/N(%) | 7/11 (63.6%) | 25/37 (67.6%) | 10/15 (66.7%) |  |  |
| **Days in hospital (N=49)** | 14.0 (13.5-25.5) | 20.0 (14.0-26.0) | 23.0 (16.0-30.0) | 0.351 |  |
| **Diabetes,n/N(%)** | 0/8 (0%) | 2/34 (5.9%) | 5/15 (33.3%) | 0.015 | 0.022^C^ |
| **Hypertension,n/N(%)** | 0/8 (0%) | 0/34 (0%) | 8/15 (53.3%) | <0.001 | 0.019^B^ |
|  |  |  |  |  | <0.001^C^ |
| **Cardiovascular disease,n/N(%)** | 0/8 (0%) | 0/34 (0%) | 4/15 (26.7%) | 0.011 | 0.006^C^ |
| **Chronic liver disease ,n/N(%)** | 0/8 (0%) | 1/34 (2.9%) | 1/15 (6.7%) | 1.000 |  |
| **Operation history,n/N(%)** | 0/8 (0%) | 10/34 (29.4%) | 6/15 (40.0%) | 0.133 |  |
| **Signs and symptoms** |  |  |  |  |  |
| Cough,n/N(%) | 1/8 (12.5%) | 25/31 (80.6%) | 14/15 (93.3%) | <0.001 | 0.001^A^ |
|  |  |  |  |  | <0.001^B^ |
| Pharyngalgia,n/N(%) | 2/8 (25.0%) | 6/31 (19.4%) | 0/15 (0%) | 0.180 |  |
| Sputum production,n/N(%) | 0/8 (0%) | 17/31 (54.8%) | 11/15 (73.3%) | 0.002 | 0.013^A^ |
|  |  |  |  |  | 0.001^B^ |
| Haemoptysis,n/N(%) | 0/8 (0%) | 0/31 (0%) | 1/15 (6.7%) | 0.426 |  |
| Dyspnoea,n/N(%) | 1/8 (12.5%) | 2/31 (6.5%) | 7/15 (46.7%) | 0.007 | 0.003^C^ |
| Thoracalgia,n/N(%) | 0/8 (0%) | 2/31 (6.5%) | 2/15 (13.3%) | 0.610 |  |
| Myalgia or fatigue,n/N(%) | 1/8 (12.5%) | 9/31 (29.0%) | 10/15 (66.7%) | 0.012 | 0.027^B^ |
|  |  |  |  |  | 0.025^C^ |
| Headache,n/N(%) | 2/8 (25.0%) | 5/31 (16.1%) | 3/15 (20.0%) | 0.888 |  |
| Diarrhoea,n/N(%) | 3/8 (37.5%) | 11/31 (35.5%) | 5/15 (33.3%) | 1.000 |  |
| Highest temperature,℃  (N=52) | 37.35 (36.48-38.73) | 37.80  (37.30-39.10) | 38.30  (37.85-39.05) | 1.000 |  |
| Systolic pressure,mmHg  (N=50)  **Supplementary table 2. Age-related demographics and baseline characteristics of patients with SARS-CoV-2 infection.**  **Abbreviations:** SARS-CoV-2, severe acute respiratory syndrome coronavirus 2; IQR, interquartile range; Data are median (IQR) or n/N (%), where N is the total number of patients with available data. *P* values comparing the difference among three groups are from Kruskal-Wallis test. Differences comparing between two groups are from *χ*^2^ test, Fisher’s exact test, or Mann-Whitney U test. A, B, C mean the *P* value of comparison between group Age≤ 18 and 19 ≤Age≤ 59, Age≤ 18 and Age ≥60, 19 ≤Age≤ 59 and Age≥ 60, respectively. | 123 (99-133) | 121 (110-135) | 133 (124-148) | 0.026 | 0.029^C^ |
