# Supplementary figures and images for: The Characterization of Disease Severity Associated IgG Subclasses Response in COVID-19 Patients
Source: Front Immunol. 2021 Mar 4;12:632814. doi: 10.3389/fimmu.2021.632814 (PMC7982848; doi:10.3389/fimmu.2021.632814)

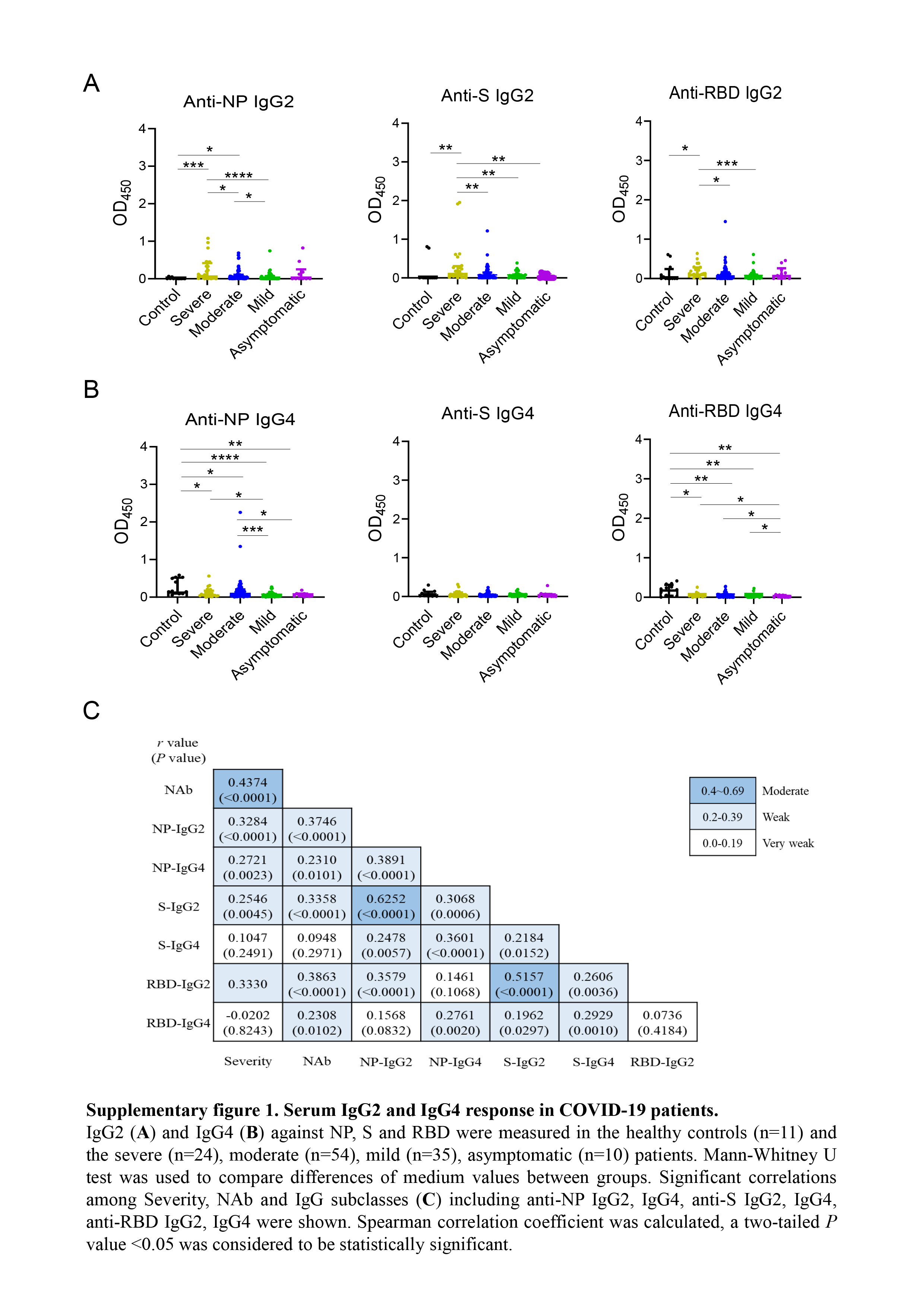

Supplement: Supplementary file 3 [file Image_1.TIF]

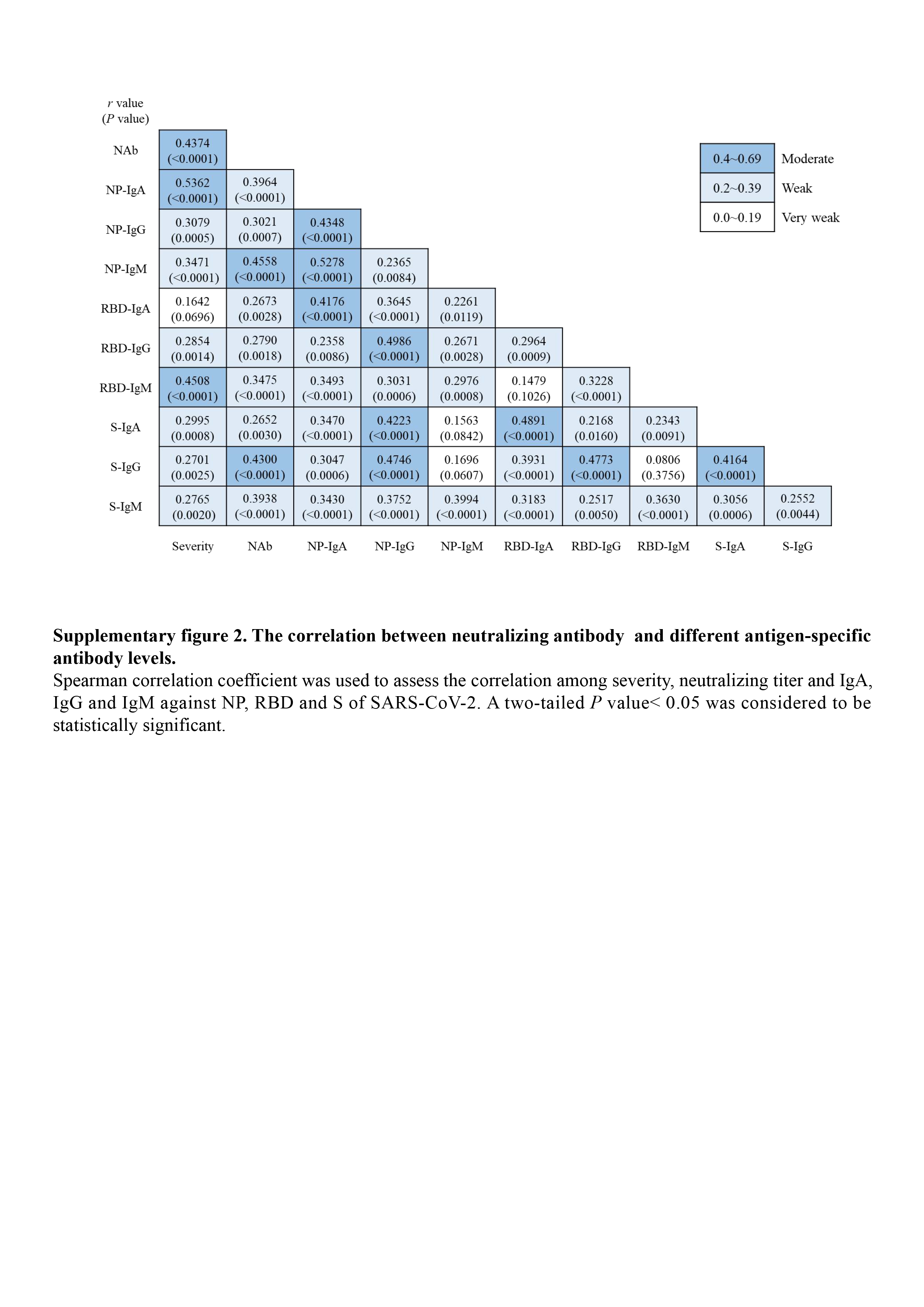

Supplement: Supplementary file 4 [file Image_2.TIF]

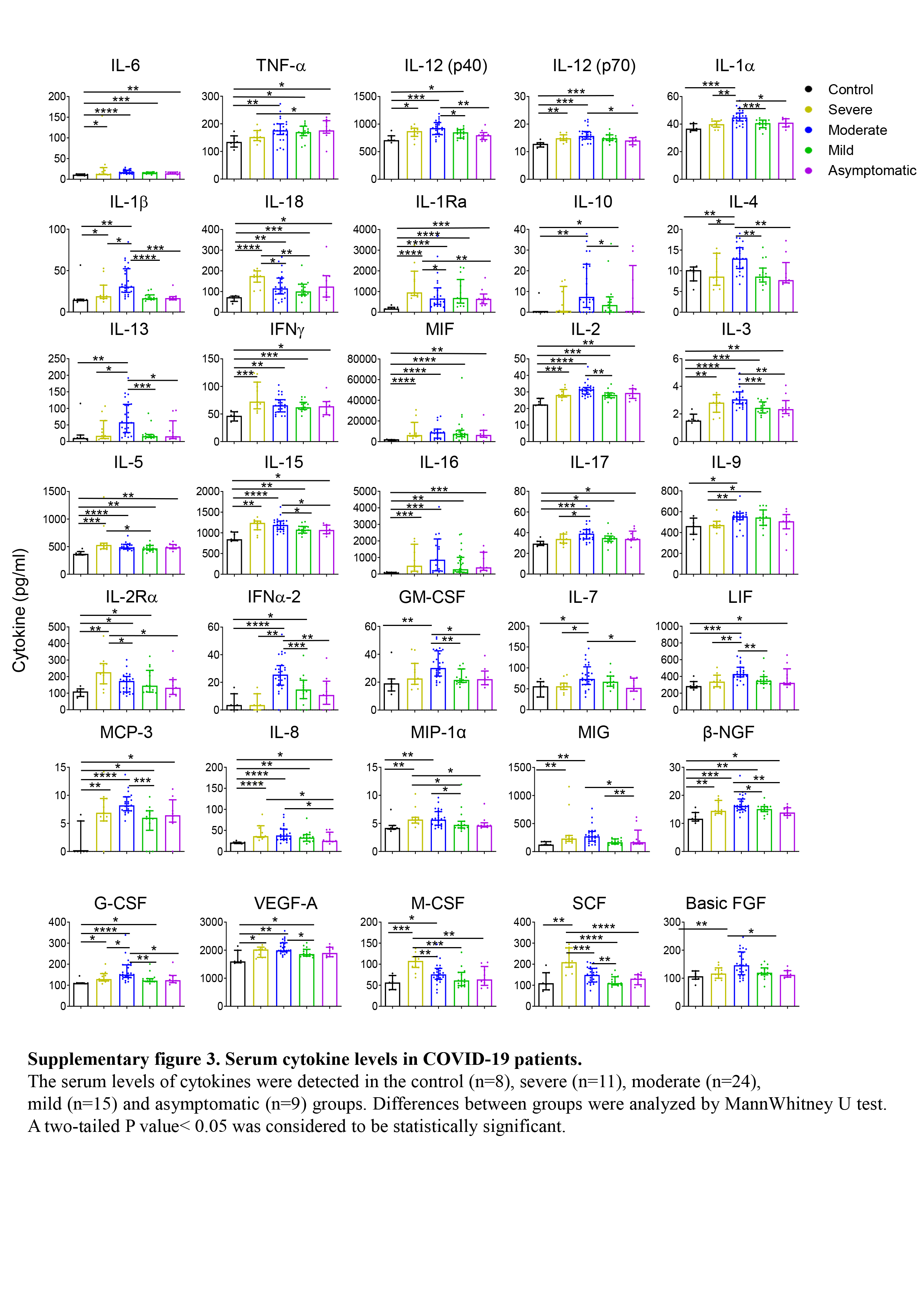

Supplement: Supplementary file 5 [file Image_3.TIF]

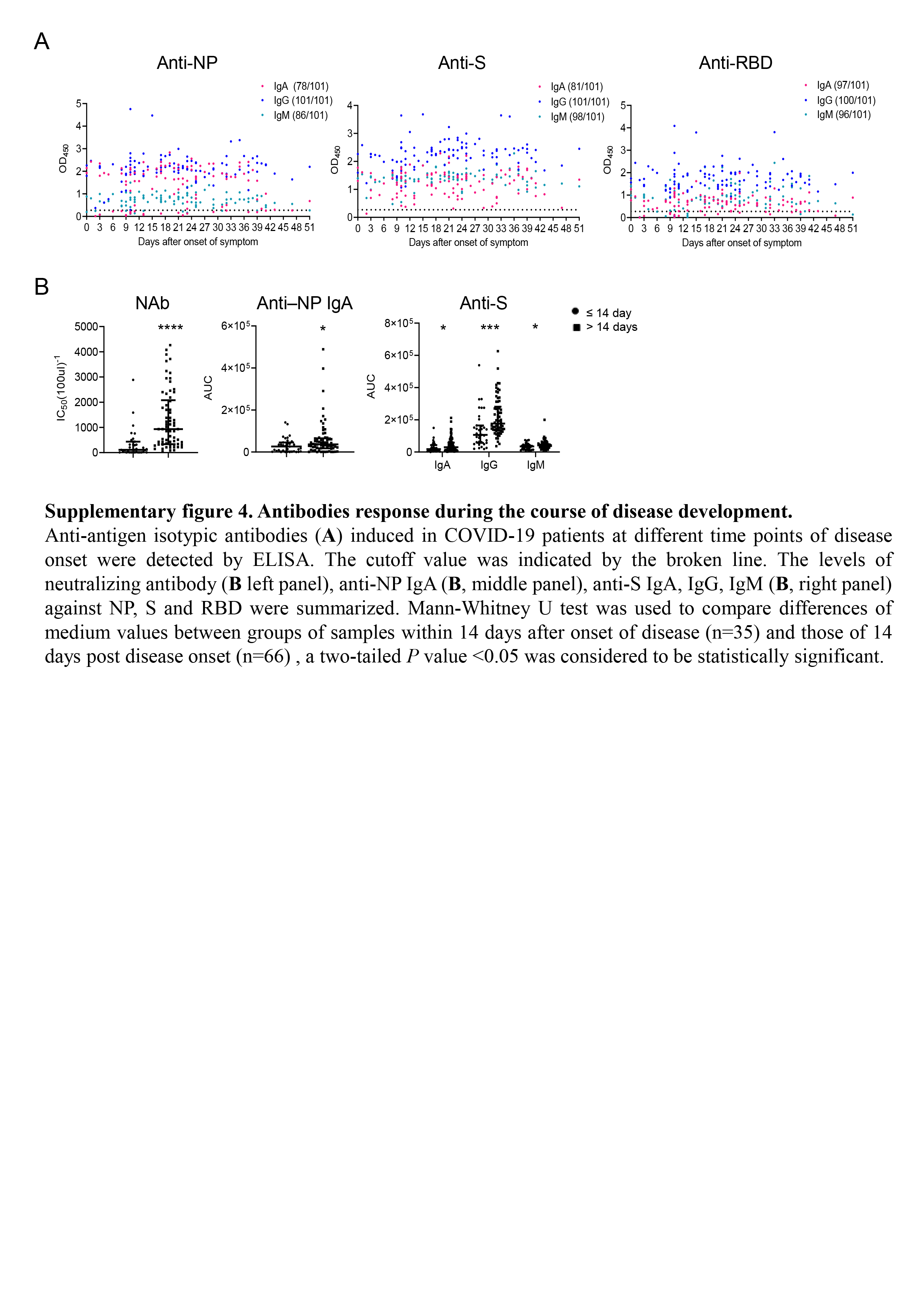

Supplement: Supplementary file 6 [file Image_4.TIF]

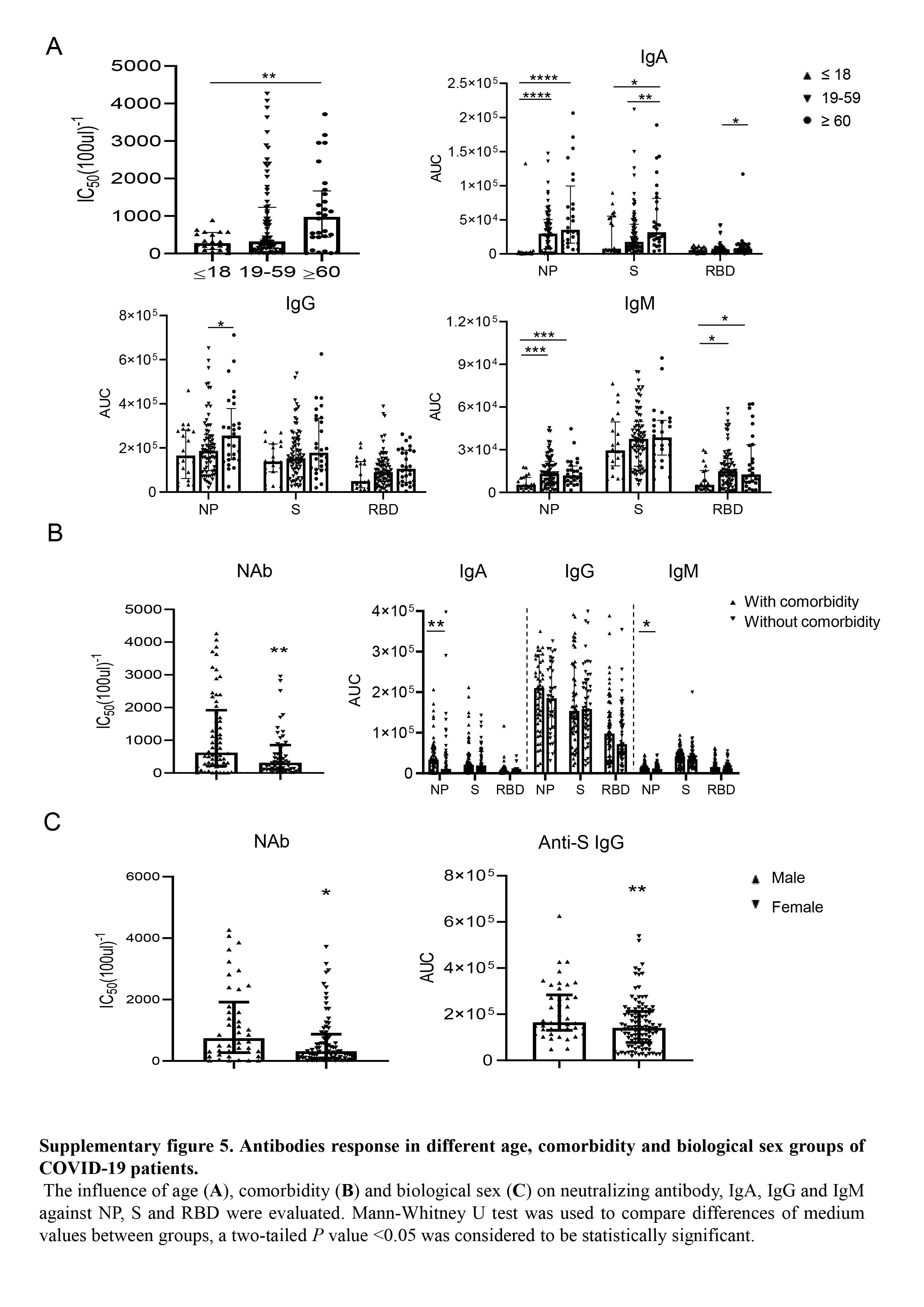

Supplement: Supplementary file 7 [file Image_5.jpg]
